# Genome Sequence Analysis of *Staphylococcus equorum* Bovine Mastitis Isolate UMC-CNS-924

**DOI:** 10.1128/genomeA.00840-13

**Published:** 2013-10-17

**Authors:** Michael J. Calcutt, Mark F. Foecking, Hsin-Yeh Hsieh, Jeanette Perry, George C. Stewart, John R. Middleton

**Affiliations:** Department of Veterinary Pathobiology, University of Missouri, Columbia, Missouri, USAa; Bond Life Science Center, University of Missouri, Columbia, Missouri, USAb; Department of Veterinary Medicine and Surgery, University of Missouri, Columbia, Missouri, USAc

## Abstract

Intramammary infections in dairy cattle are frequently caused by staphylococci, resulting in mastitis and associated economic losses. A draft genome sequence was determined for *Staphylococcus equorum* UMC-CNS-924, isolated from the milk of a Holstein cow, to better understand the genetic basis of its pathogenesis and adaptation to the bovine mammary gland.

## GENOME ANNOUNCEMENT

Historically, staphylococcal species other than *Staphylococcus aureus* have been considered to be relatively minor causes of bovine mastitis, but recent studies have recognized the increasing role of additional taxa (1, 2). Relatively little is known about the pathogenomics of *Staphylococcus equorum*. Accordingly, a draft genome sequence of *S. equorum* was determined to allow for comparative analyses of host adaptation and genome evolution within this well-studied genus of the *Firmicutes*.

Strain UMC-CNS-924 was isolated in March 2006 from the milk of a Holstein cow at the University of Missouri Foremost Dairy Center (under IACUC approval). This organism was recovered in pure culture at a titer of >1,000 CFU/ml of milk from a single mammary quarter. This isolate was selected for further study, as it was associated with only a mild inflammatory response in the infected gland, possibly suggesting host adaptation (somatic cell count, 11,000 cells/ml; reference range, ≤200,000 cells/ml).

Genomic DNA from *S. equorum* UMC-CNS-924 was sequenced by 454 Titanium sequencing of fragment libraries at the Genome Institute of Washington University, St. Louis, MO. A total of 316,668 sequence reads were assembled *de novo* using Newbler software (Roche), resulting in a draft genome of 2,700,865 bp, with 46× coverage depth, contained within 39 contigs. The contig N_50_ is 197.7 kb and the largest contig is approximately 375 kb. The G+C content is 32.9%. Annotation of the contigs was performed by using the PGAAP pipeline at the National Center for Biotechnology Information, resulting in the delineation of 2,605 open reading frames (ORFs) and 57 tRNAs. Five rRNA-encoding operons are predicted to be present based on BLASTn analysis of the contig termini.

The initial comparative analysis to the publicly available genome sequences of *S. equorum* strain Mu2 (3) and the closely related strain *Staphylococcus* sp. OJ82 (4) demonstrated very high degrees of synteny within corresponding contigs but multiple insertions of additional integrative elements in the genomes in strains Mu2 and OJ82. This strain-variable incursion accounted for much of the ~200-kb difference in the genome size between strain UMC-CNS-924 and both strains Mu2 and OJ82. No clustered regularly interspaced short palindromic repeat (CRISPR) units were identified in any of the three strains.

An analysis of the potential mobile genetic elements in strain UMC-CNS-924 revealed four contigs that represented multicopy plasmid replicons (subsequently confirmed to be in circular configuration by inverse PCR). The smallest plasmid (2.3 kb) was identical to the lincomycin resistance-conferring plasmid pLNU1, originally identified in a *Staphylococcus chromogenes* isolate from a subclinical bovine mastitis case in Germany (5). One plasmid, designated pSEQU3 (4.8 kb), harbors a tetracycline resistance determinant and has only two differences from a putative replicon in the contig set of *Staphylococcus vitulinus* F1028 (6). Resistance determinants were not identified on plasmids pSEQU1 (15.6 kb) or pSEQU2 (8.8 kb).

The availability of this data set affords comparative genomic approaches to identify features shared by, and potentially transferred between, staphylococci that cause bovine mastitis, and it allows for a comparison of possible differences in niche colonization and inflammatory sequelae.

### Nucleotide sequence accession numbers.

This whole-genome shotgun project has been deposited at DDBJ/EMBL/GenBank under the accession no. AVBD00000000. The version described in this paper is version AVBD01000000.

## References

[1] Piessens V Van Coillie E Verbist B Supré K Braem G Van Nuffel A De Vuyst L Heyndrickx M De Vliegher S 2011 Distribution of coagulase-negative *Staphylococcus* species from milk and environment of dairy cows differs between herds. J. Dairy Sci. 94:2933–2944 21605763 10.3168/jds.2010-3956

[2] Piessens V De Vliegher S Verbist B Braem G Van Nuffel A De Vuyst L Heyndrickx M Van Coillie E 2012 Intra-species diversity and epidemiology varies among coagulase-negative *Staphylococcus* species causing bovine intramammary infections. Vet. Microbiol. 155:62–71 21889271 10.1016/j.vetmic.2011.08.005

[3] Irlinger F Loux V Bento P Gibrat JF Straub C Bonnarme P Landaud S Monnet C 2012 Genome sequence of *Staphylococcus equorum* subsp. *equorum* Mu2, isolated from a French smear-ripened cheese. J. Bacteriol. 194:5141–5142 22933766 10.1128/JB.01038-12 PMC3430327

[4] Sung JS Chun J Choi S Park W 2012 Genome sequence of the halotolerant *Staphylococcus* sp. strain OJ82, isolated from Korean traditional salt-fermented seafood. J. Bacteriol. 194:6353–6354 23105083 10.1128/JB.01653-12 PMC3486393

[5] Lüthje P von Köckritz-Blickwede M Schwarz S 2007 Identification and characterization of nine novel types of small staphylococcal plasmids carrying the lincosamide nucleotidyltransferase gene *lnu(A)*. J. Antimicrob. Chemother. 59:600–606 17329268 10.1093/jac/dkm008

[6] Nam YD Chung WH Seo MJ Lim SI 2012 Draft genome sequence of *Staphylococcus vitulinus* F1028, a strain isolated from a block of fermented soybean. J. Bacteriol. 194:5961–5962 23045483 10.1128/JB.01332-12 PMC3486121

